# Radiological characteristics of hepatocellular carcinoma that achieved complete response after chemoembolization with drug-eluting beads for liver transplantation planning

**DOI:** 10.31744/einstein_journal/2023AO0307

**Published:** 2023-10-18

**Authors:** Leonardo Guedes Moreira Valle, Marcela Juliano Silva Cunha, Bruno Pagnin Schmid, Priscila Mina Falsarella, Marcelo Bruno de Rezende, Guilherme Eduardo Gonçalves Felga, Renata Emy Ogawa, Rodrigo Gobbo Garcia, Breno Boueri Affonso, Felipe Nasser, Francisco Leonardo Galastri

**Affiliations:** 1 Hospital Israelita Albert Einstein São Paulo SP Brazil Hospital Israelita Albert Einstein, São Paulo, SP, Brazil.

**Keywords:** Living donors, Liver neoplasms, Carcinoma, hepatocellular, Chemoembolization, therapeutic, Neoadjuvant therapy, Response evaluation criteria in solid tumors, Radiology, interventional

## Abstract

**Objective:**

To describe the radiological characteristics of hepatocellular carcinoma (HCC) lesions that achieved a complete response following drug-eluting bead transarterial chemoembolization (DEB-TACE) preceding liver transplantation.

**Methods:**

This single-center case-control study enrolled patients with hepatocellular carcinoma who underwent neoadjuvant DEB-TACE therapy, were followed up with contrast-enhanced magnetic resonance imaging or computed tomography, and were successively evaluated according to the modified Response Evaluation Criteria in Solid Tumors. The HCCs were divided into two groups based on their diameter (Group A: ≤3cm; Group B: 3cm). Viability was assessed using the Kaplan-Meier method according to tumor size categories. The relationship between tumor variables was analyzed using bivariate Cox regression.

**Results:**

Three-hundred and twenty-eight patients with 667 hepatocellular carcinomas who underwent their first DEB-TACE session were enrolled. A total of 105 hepatocellular carcinomas in 59 patients exhibited complete response after the initial DEB-TACE session and were divided into Group A (92 HCCs) and Group B (13 HCCs). The diameter in Group A decreased significantly compared to the pre-procedure size until the second assessment (p<0.001), with no subsequent reduction in diameter, despite maintaining a complete response. In Group B, the reduction in diameter remained significant compared with the initial value until the sixth imaging evaluation (p=0.014). The average reduction was 45.1% for Group B and a maximum of 14.9% in Group A.

**Conclusion:**

HCCs >3cm exhibited a greater reduction in size and a longer time to recurrence. HCCs ≤3cm had a shorter relapse time. The recurrence rates were similar. These findings may aid in planning for liver transplantation.

## In Brief

Valle et al. explored the radiological characteristics of hepatocellular carcinoma that achieved complete response after chemoembolization with drug-eluting beads. Patients were followed up and contrast-enhanced magnetic resonance imaging or computed tomography were performed. Patients were successively evaluated according to the modified Response Evaluation Criteria in Solid Tumors. These findings may aid in planning for liver transplantation.

## Highlights

Once complete hepatocellular carcinoma necrosis is achieved, the diameter of the hepatocellular carcinoma decreases significantly, regardless of its initial size.

Hepatocellular carcinoma >3cm showed greater reduction and longer time to recurrence.

Hepatocellular carcinoma ≤3cm exhibited a shorter time to relapse.

Recurrence rates were similar, as long as complete response was achieved.

## Visual Abstract

**Figure:**
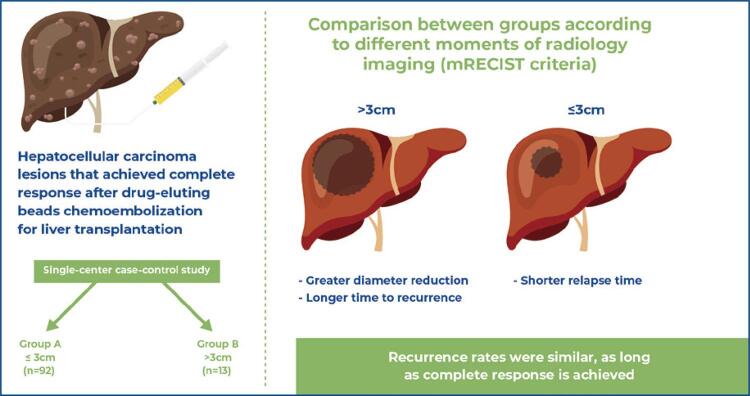

## INTRODUCTION

Hepatocellular carcinoma (HCC) is the most common primary liver cancer and the third leading cause of cancer-related mortality worldwide, with an increasing incidence and mortality in North America and several European regions.^(1-4)^ At diagnosis, most patients present at an advanced stage for which curative treatment is not possible.^(4,5)^

Liver transplantation (LT), tumor resection, and radiofrequency ablation are curative treatment modalities for HCC.^(4,5)^ As such, locoregional therapies play a key role in the pre-transplantation scenario.^(4-6)^

Transarterial chemoembolization (TACE) is a transarterial locoregional treatment modality that is conventionally used palliatively in patients during the intermediate stage (BCLC B).^(5)^ It can also be used as a neoadjuvant method for liver disease control (bridge therapy), while patients are awaiting liver transplantation, or to help patients fulfill Milan’s criteria for transplantation (downstaging) (BCLC A, B, and C).^(5)^

Drug-eluting bead transarterial chemoembolization (DEB-TACE) is a chemoembolization modality has a standardized technique, is associated with fewer side effects, and reports better post-transplant recurrence-free survival.^(7-9)^

The most commonly used method to evaluate the radiological response of solid tumors after locoregional therapy is the modified Response Evaluation Criteria in Solid Tumors (mRECIST), which defines complete response (CR) as the best radiological response possible, wherein there is no vascular enhancement suggestive of tumor viability, and is correlated with improvement in overall survival (OS).^(10)^

The radiological characteristics of HCCs that achieved CR after locoregional therapy remains unclear. The impact of the initial tumor size on the time to relapse, maximum diameter reduction, and recurrence rates need to be explored.^(11)^

This information is essential for patients undergoing neoadjuvant therapy, to better indicate DEB-TACE therapy in the pre-transplantation scenario based on real perspectives regarding HCC diameter reduction for downstaging and to establish better standards of radiological follow-up based on the average tumor recurrence time for patients undergoing bridge therapy. This could also potentially decrease the rate of exclusion from the liver transplantation waiting list (dropouts).

## OBJECTIVE

To describe the radiological characteristics of hepatocellular carcinoma in patients undergoing neoadjuvant DEB-TACE for liver transplantation who achieved a complete response, and to establish standards for better patient selection for DEB-TACE as neoadjuvant intervention.

## METHODS

Noninvasive HCC diagnosis was defined according to the EASL guidelines as new lesions with sizes ≥1cm and/or HCCs that increased in size between imaging examinations that present arterial phase hyperenhancement and venous or delayed phase washout on multiphasic computed tomography (CT) or magnetic resonance imaging (MRI).^(12)^ The radiological characteristics were analyzed according to the mRECIST criteria, and the same imaging method (CT or MRI) was used for all patients during follow-up.^(10)^

The inclusion criteria were HCC lesions with CR in patients with BCLC 0, A, or B from the institutional LT program, who underwent DEB-TACE for bridging or downstaging therapy. The exclusion criteria were as follows: patients with BCLC C and D; patients who did not provide consent; patients who did not undergo follow-up imaging because of loss to follow-up, death, or transplantation; patients who underwent imaging examinations outside the defined period; and lesions that did not achieve CR after the first DEB-TACE session.

This single-center case-control study was approved by the Institutional Review Board of *Hospital Israelita Albert Einstein* (# 376/2011). Written informed consent was obtained from all participants.

## Imaging protocols

A complete response of the HCC was identified as a lesion without any sign of contrast enhancement according to mRECIST. The viability of the HCCs, recurrence or maintenance of CR, and tumor diameter were assessed over time. Patients were followed-up with contrast-enhanced MRI or multiphasic CT within 30–90 days after the first DEB-TACE session (D1) and underwent successive evaluations. Radiological control was performed within 90 days of the final image evaluation: D2 = D1 + 90 days; D3 = D2 + 90 days; D4 = D3 + 90 days; D5 = D4 + 90 days; and D6= D5 + 90 days.

The HCCs were divided into two groups, according to the diagnosis imaging exam (CT or MRI) performed at 10 to 60 days preceding the procedure: Group A: HCCs ≤3cm; Group B: HCCs >3cm.

The recurrence rate and time to recurrence were assessed, as well as the dose of chemotherapy delivered to the HCC, the presence of pseudocapsules, and the serum level of alpha-fetoprotein before the procedure, and were tested as independent variables.

## Drug-eluting bead chemoembolization technique

All chemoembolizations were performed in the angiosuite of a tertiary care hospital and were led by a highly experienced interventional radiologist (more than seven years in practice) assisted by two interventional radiology fellows, with the patient in a supine position, using a Philips Azurion Clarity IQ (Philips Medical Systems, Amsterdam, The Netherlands) angiography system.

The procedures were performed under local anesthesia using 2% lidocaine, sedation, and systemic analgesia. The technique was selected after multidisciplinary discussion between the anesthetist and interventional radiology team.

The intervention was performed using a common femoral artery approach. Superior mesenteric artery, celiac trunk, and common hepatic artery angiograms were obtained using a 5-F Cobra 2 or Simmons 2 catheter (Cordis, Bridgewater, New, USA) to outline the hepatic artery anatomy, delineate the tumor, identify its feeding vessels, and confirm portal vein patency.

The feeding vessels supplying the tumor were catheterized with a 2.8-F microcatheter (Progreat; Terumo, Tokyo, Japan), and the tumors were embolized with a fluoroscopic guided injection of iodinated contrast medium mixed with one vial of 100–300µm DEBs (DC Bead; Biocompatibles, Farnham, United Kingdom) or 50–100µm HepaSphere (Merit Medical Systems, United States) loaded with 50mg of doxorubicin.

The embolization endpoint was defined as near stasis observed in the artery directly feeding the tumor. In target lesions that presented with vascular lake phenomenon, additional embolization was performed using calibrated microspheres 300-500µm (Bead Block, Biocompatibles, UK) or microspheres 355–500µm (Contour, Boston, USA) until the endpoint was achieved. A suturing device (Perclose Proglide, Abbott, United States) was used for access closure in all patients.

After chemoembolization, all patients remained at a post-anesthetic recovery unit for 6 hours, with the lower limbs at rest to prevent arterial access complications. The patients were discharged from the hospital on the same day as the procedure, after the Interventional Radiology team, who performed the procedure clinically evaluated the lower extremity pulses, puncture site, and symptoms related to hepatic embolization.

## Statistical analysis

Necrosis and tumor sizes were described according to the groups throughout the different periods of radiology imaging (D1, D2, D3, D4, D5, and D6). The mean and standard deviation were assessed and compared using generalized estimation equations with normal distribution and identity link function, assuming an autoregressive correlation^(1)^ correlation matrix between evaluations, followed by the Bonferroni multiple comparison method to assess each group and the times at which the differences occurred.

Viability time was assessed using the Kaplan–Meier method according to tumor size categories, and the relationship between the previous tumor variables was analyzed using bivariate Cox regression. Statistical analyses were performed by a biomedical statistician using SPSS software (version 22.0; IBM, Armonk, NY, USA), and statistical significance was set at 5%.

## RESULTS

From April 2011 to June 2016, 328 patients with 667 HCCs underwent their first DEB-TACE session. Among these 667 HCCs, 510 were considered target lesions. Thirty-four patients with 80 HCCs were excluded due to a lack of follow-up imaging.

Therefore, 430 patients with HCCs were included in this study. A total of 105 HCCs in 59 patients (men: 49, women: 10; mean age: 58.2±6.5 [range: 43–72] years) achieved CR after the first DEB-TACE session.

**Figure 1:**
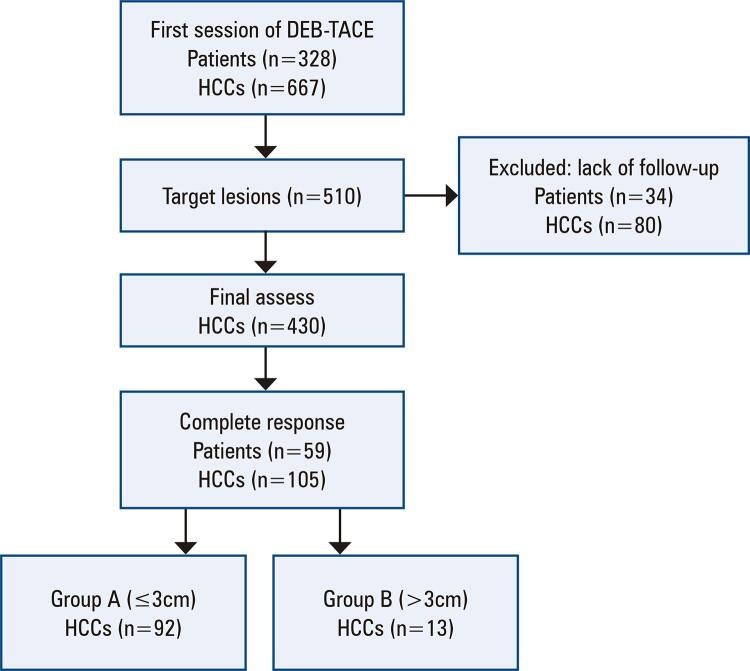
Patient flow chart

DEB-TACE: drug-eluting beads chemoembolization; HCC: hepatocellular carcinomas

**Figure 2:**
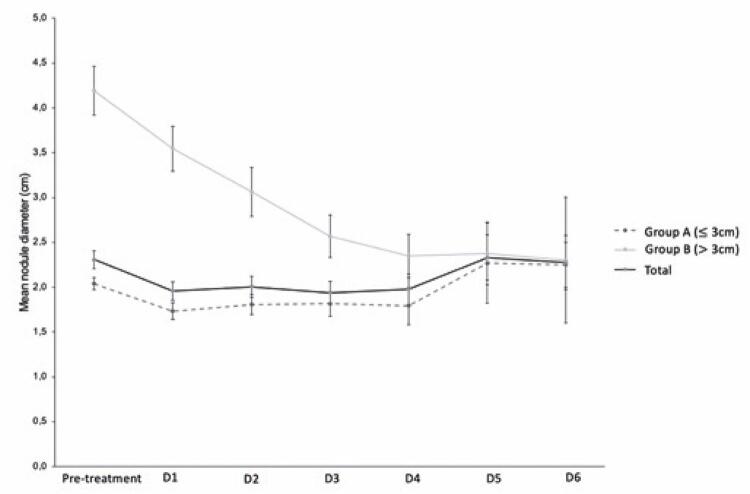
Showing mean tumors sizes according to the groups throughout different moments of radiology imaging (pre-treatment, D1, D2, D3, D4, D5 and D6)

Finally, 92 tumors were ≤3cm in diameter and 13 tumors were >3cm in size (Figure 1). The mean tumor sizes before and after treatment are summarized in table 1 and figure 2.

**Table 1:** Mean tumor sizes of groups (HCC ≤3cm and HCC >3cm) over time (pre-treatment, D1, D2, D3, D4, D5, and D6) based on radiologic imaging

Time	Group A (HCC ≤3cm)		Group B (HCC >3cm)		Total	
		
	n	Diameter (Mean±SD)	n	Diameter (Mean±SD)	n	Diameter (Mean±SD)
Pre-treatment	92	2.039±0.645	13	4.192±0.993	105	2.306±0.993
D1	92	1.734±0.869	13	3.546±0.903	105	1.958±1.056
D2	59	1.807±0.863	11	3.064±0.907	70	2.004±0.978
D3	31	1.816±0.777	6	2.567±0.575	37	1.938±0.792
D4	12	1.792±0.725	6	2.35 ±0.589	18	1.978±0.718
D5	3	2.267±0.777	4	2.375±0.69	7	2.329±0.668
D6	2	2.25±0.354	2	2.3±0.990	4	2.275±0.608

Diameters are expressed in centimeters.

HCC: hepatocellular carcinomas; SD: standard deviation.

In Group A, the reduction in diameter was significant compared to the pre-procedure size until the second assessment (D2), with a maximum mean reduction of 0.4cm, with no subsequent reduction in diameter, despite maintaining 100% necrosis (Table 2). In Group B, the size reduction was significant compared to the initial value until the sixth assessment (D6), achieving a maximum mean reduction of 1.83cm (Table 3).

**Table 2:** Showing diameter reduction in Group A (HCC ≤3cm)

Time	Estimated mean difference	Standard error	p value	Confidence interval 95%
D1	0.31	0.05	<0.001	0.12; 0.49
D2	0.40	0.08	<0.001	0.12; 0.68
D3	0.34	0.11	0.294	-0.06; 0.73
D4	0.31	0.17	>0.999	-0.29; 0.90
D5	0.09	0.32	>0.999	-1.00; 1.19
D6	-0.21	0.43	>0.999	-1.69; 1.28

The estimated mean difference was calculated by comparing the mean diameter during the pre-treatment period and the mean diameters at different moments of radiology imaging (D1, D2, D3, D4, D5, and D6).

**Table 3:** Diameter reduction in Group B (HCC >3cm)

Time	Estimated mean difference	Standard error	p value	Confidence interval 95%
D1	0.65	0.14	0.001	0.15; 1.14
D2	1.09	0.20	<0.001	0.39; 1.78
D3	1.45	0.27	<0.001	0.51; 2.38
D4	1.70	0.31	<0.001	0.63; 2.77
D5	1.83	0.36	<0.001	0.57; 3.08
D6	1.76	0.46	0.014	0.15; 3.37

The estimated mean difference was calculated by comparing the mean diameter during the pre-treatment period and the mean diameters at different moments of radiology imaging (D1, D2, D3, D4, D5, and D6).

Hepatocellular carcinoma measuring >3cm showed a greater reduction in diameter. The average percentage reduction was 45.1% in Group B (D6) and 14.9% in Group A (D1). From the fourth imaging assessment (D4), there was no difference in diameter between the two groups (p=0.035) (Table 4).

**Table 4:** Comparison between groups A (HCC ≤3cm) and B (HCC >3cm) over time (pre-treatment, D1, D2, D3, D4, D5 and D6) based on radiologic imaging

Time	Mean difference	Standard error	p value	Confidence interval 95%
Pre-treatment	-2.15	0.24	<0.001	-2.97; -1.33
D1	-1.81	0.24	<0.001	-2.63; -0.99
D2	-1.47	0.25	<0.001	-2.32; -0.61
D3	-1.04	0.29	0.035	-2.05; -0.03
D4	-0.76	0.34	>0.999	-1.93; 0.41
D5	-0.42	0.46	>0.999	-2.01; 1.17
D6	-0.19	0.61	>0.999	-2.29; 1.92

**Figure 3:**
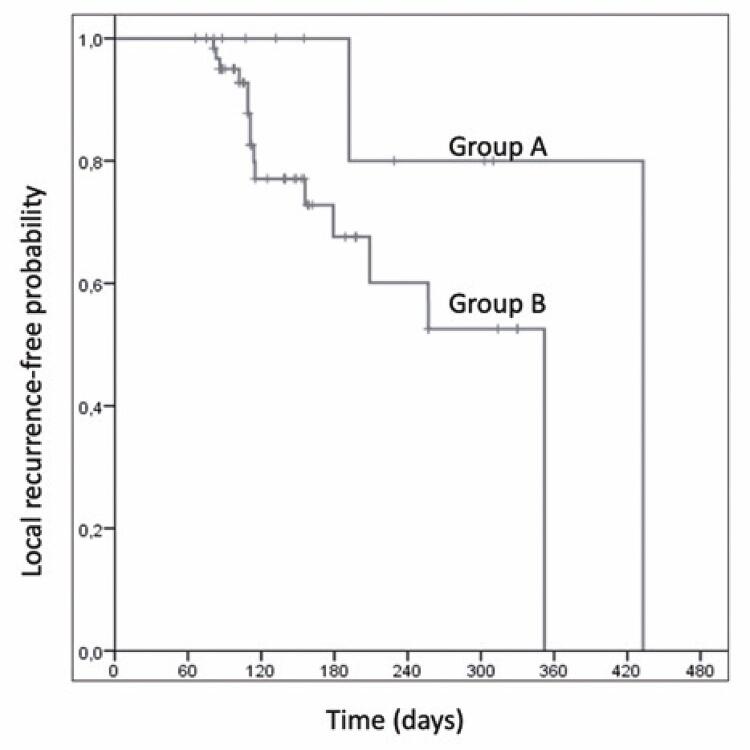
Kaplan–Meier curves showing local recurrence of tumors in Groups A (HCC ≤3cm) and B (HCC >3cm) after complete necrosis

Although there was no statistical difference (p=0.124) in the local recurrence of tumors between the groups after CR, the time to recurrence in Group B was 120 days longer than in Group A (Figure 3).

Group A (HCC ≤3cm) and B (HCCs >3cm).

There was no significant association in time of recurrence regarding the presence of pseudocapsule (HR= 0.621; 95%CI= 0.125–3.079; p=0.559), serum level of alpha fetoprotein (HR= 1.002; 95%CI= 0.95–1.02; p=0.28), and the dose of chemotherapy delivered by HCC (HR= 0.98; 95%CI; 0.998–1.007; p=0.326).

## DISCUSSION

The results revealed that when CR was achieved in both groups post-DEB-TACE, there was a significant reduction in the lesion diameter in the first post-DEB-TACE image. Therefore, it is possible to infer that once CR is achieved, regardless of whether the patient is in the bridge and downstaging stages, there will be a reduction in the HCC size.

However, this study also showed that the HCC size significantly influenced the radiological characteristics and must be considered as a parameter for an efficient follow-up protocol and better LT planning.

When comparing the relative and absolute tumor size reductions between the groups over the evaluated periods, the reduction in diameter was greater in Group B than in Group A, and the HCCs in Group A exhibited a shorter time to relapse.

It is possible to infer that smaller HCCs, especially those <2cm, might stop reducing in diameter earlier than Group B HCCs, as they are usually dysplastic HCCs and have increased portal blood supply and different hemodynamics (preferential arterial flow to the larger tumor).

Regarding HCCs >3cm, a significant reduction in diameter was observed relative to their original size up to the D6 interval. This finding might help predict the success of locoregional treatment for bridging and downstaging tumors that become nonviable after the first DEB-TACE procedure.

Knowledge of the behavior of this group (HCC >3cm) is especially important because in patients with multiple HCCs, with one above 3cm, it is necessary to reduce the axial diameter to fit the transplant criteria.^(13)^The significant reduction in the diameter of the HCCs observed in this group reinforces the indication for downstaging and supports all efforts to provide DEB-TACE to this patient population.

Although the number of HCCs for analysis in this group was limited owing to the difficulty of achieving CR after the first DEB-TACE, the larger the tumor diameter, once CR is reached, a reduction in diameter can be expected for the entire period as long as there is no local recurrence.

A contradictory result was the higher mean diameter in the fifth and sixth assessments for Group A. This finding can be explained by the dropout rate of patients due to successful LT during follow-up. As a result, it is assumed that the remaining HCCs may represent a subgroup with a poor prognosis (larger tumor size, higher recurrence rates, and development of other LT contraindications).

Another key point of this study concerns the relationship between recurrence and tumor size. The recurrence times were not statistically significant between the groups, but the time to recurrence was 120 days longer in Group B. This difference in relapse time, although small, may be important in regions with longer waiting times for LT, such as Brazil, which has an average waiting time of almost three months.^(14)^

Finally, the other analyzed parameters (chemotherapy dose, presence of pseudocapsules, and serum alpha-fetoprotein level) did not influence size reduction or recurrence.

A previous study reported that macrovascular invasion is a predictor of early HCC recurrence, whereas diabetes mellitus is a predictor of late HCC recurrence in patients with CR.^(11)^ These parameters were not analyzed in the present study, but should also be considered as a tailored post-TACE follow-up strategy.

Previous reports have shown that the presence of a pseudocapsule and the addition of a chemoembolic agent are of paramount importance in the radiological response in HCC, increasing the percentage of tumor necrosis following DC-BEAD in neoadjuvant treatment before LT.^(15)^ One hypothesis for the disparities shown in the present study is that once CR is reached, these variables may have little influence on the subsequent evolution of HCC.

The limitations of this study include its single-center analysis and small sample size (despite all efforts, 34 patients with 80 HCCs were excluded because they were lost to follow-up).

## CONCLUSION

Once complete hepatocellular carcinoma necrosis is achieved, there will be a significant reduction in the diameter of the hepatocellular carcinoma, regardless of its initial size. Hepatocellular carcinoma >3cm exhibited a greater reduction and a longer time to recurrence. Hepatocellular carcinoma ≤3cm had a shorter time to relapse. Recurrence rates are similar as long as complete response is achieved. These findings may help predict locoregional treatment responses and aid in liver transplantation planning.
